# The Care and Management of Premature Infants

**Published:** 1932

**Authors:** Francis J. Hector

**Affiliations:** Assistant Obstetric Physician and Gynœcologist, Bristol General Hospital


### THE CARE AND MANAGEMENT OF PREMATURE INFANTS.

BY

FRANCIS J. HECTOR, M.D., F.R.C.S.,
*Assistant Obstetric Physician and Gynæcologist, Bristol General Hospital.*

---

DURING the last few years the care and management of premature infants has acquired additional importance, and has been more extensively studied in all civilized countries. This has been largely due to the widespread adoption of well-organized ante-natal treatment, and considerably more inductions of labour are now performed in the interests of the mother or the child.

Apart from the usual desire to rear a premature infant, however unpromising at birth, various family or domestic reasons may make its survival a matter of supreme importance. The death of the premature infant of an elderly primipara where labour has been induced, shall we say, on account of severe toxæmia, may be in the nature of stark tragedy to its parents. Furthermore, in every case the mother has had perhaps thirty-four weeks of pregnancy to endure, with all its trials and discomforts, and has every reason to expect a live baby as her reward.

It has been alleged by some that the rearing of premature infants is not, from a purely economic or sociological standpoint, worth the enormous amount of care entailed, and that such infants grow up weak and rather useless individuals, who may eventually become a charge on their fellows. Recent investigations in Germany, however, have shown that premature children, having survived the first few critical weeks, have just as much chance of becoming good workers, thinkers or athletes as any other type of child.

The two most important points in the management of premature infants are, of course, temperature regulation and feeding. The regulation of temperature is the first and constant difficulty. Even the full-time infant's tenacity of life must sometimes be sorely tested, considering the circumstances which not infrequently obtain at its birth during the winter-time. Projected into a chilly room or labour ward, seized and subjected to even dangerous solicitude, its temperature must often fall to an alarming degree, and this at a time when important anatomical changes and delicate metabolic readjustments are commencing. It may also be pointed out that by reason of its own metabolic activities the temperature of the fœtus in utero is 0·5° higher than that of its mother. Small wonder is it, therefore, that numbers of premature infants are certified as dying of "prematurity" or "congenital debility" during the first few days of life, when their deaths were really due to the effects of cold sustained immediately after delivery. Once the temperature has been allowed to fall for even a very short time in the case of a premature infant, it is unlikely that the effects of such neglect can ever be remedied by any later treatment, however thorough. Immediately after delivery, and before the cord is tied, the infant must be wrapped in a warm blanket. Tying the cord and weighing must be accomplished with the utmost dispatch, and the infant is then wrapped in gamgee, covered with blankets and put into the cot with three rubber hot-water bottles at 160°, or better still with an electric blanket. For hospital or nursing home practice the latter is a very desirable article. It costs about thirty shillings, with an additional six shillings for a waterproof cover, and can be obtained from any surgical stores. It can be used off any voltage and has a regulator switch for three temperatures, 100°, 130°, 160°. The 160° temperature should be used. The electric blanket is free from danger, and should be used arched over the infant between the enveloping blanket and the ordinary covering blankets. Excellent results have been obtained with it at Queen Charlotte's Hospital during the past two years. Immediately after reaching the ward the rectal temperature must be taken with a subnormal thermometer with a low limit of 88°. All other thermometers are quite useless for premature infants. Unless great care is taken an infant may have a rectal temperature of 90° ten minutes after delivery, although wrapped up quickly in blankets. If the infant is below 4 lb. the rectal temperature should be taken 2-hourly by day and night for a fortnight, and 4-hourly if between 4 lb. and 5 lb. It is absolutely essential to keep the temperature between 97° and 99°. Failure to do this must be put down to inefficiency on the part of the nurse. The electric blanket should be kept on at 160° day and night for a fortnight in the case of a very small baby. Good results can be obtained with an ordinary cot and hot-water bottles if sufficient care is taken. The rubber hot-water bottles should be covered with flannel or a layer of blanket.

Below a birth weight of 4 lb. no attempt at oiling or bathing must be made. No premature baby, whatever the birth weight, should be oiled without the rectal temperature having first been taken and found to be within normal limits, and even then the oiling must be done very rapidly to avoid loss of heat. In the cot the baby should lie absolutely flat with only a soft flat pillow beneath the head. The piling on of many blankets should be avoided, as they may actually weigh down the chest. A hand placed between the blankets and the baby will ensure that the former are not too heavy. Plenty of warm air should be allowed to circulate round the baby's head, over which a bonnet of cotton wool or gamgee is placed. There is a tendency to over-clothing and over-blanketing, and when placed at the bottom of a cot the infants do not get enough air. The baby's position may be changed occasionally by laying it on the opposite side.

No food should be given for the first six hours, then small sips of water, 5 per cent. glucose, or 5 per cent. glucose in saline, should be offered. To avoid dehydration sips of water should also be given between feeds. The amount and frequency of feeds is very important, and experience of extreme variations in feeds recommended in this country and abroad makes it probable that the important factor is not so much the quality as the quantity of the feeds. Gross overfeeding is the common mistake, and many premature infants are killed by too much food. Twelve hours after delivery begin with one drachm feeds 2-hourly day and night, in the case of infants over $3\frac{1}{2}$ lb. Under $3\frac{1}{2}$ lb. give $\frac{1}{2}$ drachm 2-hourly. Increase the feeds very gradually by $\frac{1}{2}$ to 1 drachm daily for the infant over $3\frac{1}{2}$ lb. and by $\frac{1}{2}$ drachm daily for the infant under $3\frac{1}{2}$ lb. The rate of increase must be determined by watching the effects of the feeds on the infant. On the slightest evidence of cyanosis or refusal to take the whole feed reduce the amount to half and give the feeds hourly for a short time, gradually reverting to 2-hourly feeds next day. Suitable feeds for a baby of $3\frac{3}{4}$ lb. would be :—

| | | | | | |
|---|---|---|---|---|---|
| Day | 1. | 1 dr. | 2-hourly. | 12 feeds. | |
| ,, | 2. | $1\frac{1}{2}$ ,, | ,, | 12 ,, | |
| ,, | 3. | 2 ,, | ,, | 12 ,, | |
| ,, | 4. | 2 ,, | ,, | 12 ,, | |
| ,, | 5. | $2\frac{1}{2}$ ,, | ,, | 12 ,, | |
| ,, | 6. | 3 ,, | ,, | 10 ,, | (omit two night feeds). |
| ,, | 7. | 3 ,, | ,, | 10 ,, | ,, ,, ,, |

After seven to ten days the infant can usually be got on to ten feeds daily, and if the weight is less than $3\frac{1}{2}$ lb. ten feeds daily will probably have to be given for a month. If the weight is over $3\frac{1}{2}$ lb., ten feeds daily should be given for two to three weeks, then eight feeds daily.

The exact amount and number of the feeds depends on :—

(1) The birth weight.

(2) How the feeds are taken. If the infant is always hungry and takes well, the amount can often be increased quite rapidly. Refusal of feeds and cyanosis are danger signs.

(3) The weight curve. If stationary after, say, ten days, increase cautiously the amount per feed.

By far the best food is breast milk, and this should always be given when possible, even if only a few drachms of colostrum or breast milk can be expressed during the first few days. The smaller and more premature the infant the more important this becomes. In maternity hospitals, where there is always a good supply of expressed breast milk available, artificial feeds are rarely given for the first three to four weeks. In private practice every attempt should be made to obtain breast milk for the infant for at least one month.

Failing breast milk, suitable artificial feeds are :—

(1) A modified milk mixture, peptonized. At Queen Charlotte's Hospital the mixture is made as follows :—

| | |
|---|---|
| Whole milk .. .. .. | 10 oz. |
| Fat whey .. .. .. | 10 ,, |
| Lactose .. .. .. | 1 ,, |
| Cream (50 per cent. fat) .. | ½ ,, |

This is peptonized for three hours. After two to three weeks the peptonization is gradually decreased by twenty minutes on subsequent alternate days.

(2) Whole milk diluted with an equal quantity of water plus 5 per cent. cane sugar.

Should any of the numerous proprietary articles be used, they should be made up in the proportions recommended on the container; but the amount of the feeds must not be as recommended, but as stated above.

The technique of feeding varies with the birth weight, the general condition of the child and its ability to suck well. The child must not be put to the breast for the first few weeks of life, especially if it is very small. A bottle with a large hole in the teat, a spoon, a medicine dropper, or a nasal catheter may be used. With small premature infants one would generally begin with a medicine dropper or pipette. The infant is not to be taken out of the cot. The hand of the nurse is placed behind its head and back and the infant gently raised up a little way from the pillow. The feed must be given at the temperature of 100° and given slowly, allowing plenty of time between the sips for the infant to swallow. When strength is gained, and the ability to suck well without getting tired, use a bottle, the teat having a large hole so that the milk flows easily. Should the child be lethargic and suck badly the feeds should be given with a nasal catheter. Rigid asepsis is necessary for this as for all methods of feeding.

The premature infant is extremely susceptible to infection, and nobody with the slightest sore throat or cold must be allowed near it. In private practice it is better to exclude everybody from the infant's room, and allow only the parents to see the child for a very short time daily for the first month, if it is very small. Should the nurse develop a cold, she should wear a face mask while attending to the child, or —better still—another nurse should take her place until the cold has gone.

The injection of whole blood or serum often saves life, apparently by conferring upon the child those antibodies which it lacks, and this especially applies to the very premature child. Excellent results have been obtained in Germany with this treatment. The blood is given intramuscularly. Any healthy donor may be used, and there is no need to group the blood. On the first day of injection 2 cc. are given, 3 cc. on the third day, 5 cc. on the fifth day, and on each subsequent alternate day give 5 cc. until ten injections have been given. The strictest aseptic precautions must, of course, be taken.

In conclusion, it may be stated that 95 per cent. of the credit for the survival of a small premature infant is due to its nurse, and a great deal has been accomplished by a nursing staff imbued with sufficient enthusiasm and determination to save even the most unpromising infants. Incredibly small infants have thus survived, and the success of some clinics with premature children weighing between one and two pounds has been remarkable.

